# Beneficial effects of *Lactobacillus reuteri 6475* on bone density in male mice is dependent on lymphocytes

**DOI:** 10.1038/s41598-019-51293-8

**Published:** 2019-10-11

**Authors:** Fraser L. Collins, Naiomy Deliz Rios-Arce, Jonathan D. Schepper, A. Daniel Jones, Laura Schaefer, Robert A. Britton, Laura R. McCabe, Narayanan Parameswaran

**Affiliations:** 1 Department of Physiology, Michigan State University, East Lansing, USA; 2 Comparative Medicine and Integrative Biology Program, Michigan State University, East Lansing, Michigan USA; 3 Department of Biochemistry and Molecular Biology, Michigan State University, East Lansing, USA; 4 Department of Chemistry, Michigan State University, East Lansing, USA; 5 Department of Molecular Virology and Microbiology, Baylor College of Medicine, Houston, USA

**Keywords:** Bacterial host response, Bone

## Abstract

Oral treatment with probiotic bacteria has been shown to prevent bone loss in multiple models of osteoporosis. In previous studies we demonstrated that oral administration of *Lactobacillus reuteri* in healthy male mice increases bone density. The host and bacterial mechanisms of these effects however are not well understood. The objective of this study was to understand the role of lymphocytes in mediating the beneficial effects of *L. reuteri* on bone health in male mice. We administered *L. reuteri* in drinking water for 4 weeks to wild type or Rag knockout (lack mature T and B lymphocytes) male mice. While *L. reuteri* treatment increased bone density in wild type, no significant increases were seen in Rag knockout mice, suggesting that lymphocytes are critical for mediating the beneficial effects of *L. reuteri* on bone density. To understand the effect of *L. reuteri* on lymphocytes in the intestinal tissues, we isolated mesenteric lymph node (MLN) from naïve wild type mice. In *ex vivo* studies using whole mesenteric lymph node (MLN) as well as CD3^+^ T-cells, we demonstrate that live *L. reuteri* and its secreted factors have concentration-dependent effects on the expression of cytokines, including anti-inflammatory cytokine IL-10. Fractionation studies identified that the active component of *L. reuteri* is likely water soluble and small in size (<3 kDa) and its effects on lymphocytes are negatively regulated by a RIP2 inhibitor, suggesting a role for NOD signaling. Finally, we show that T-cells from MLNs treated with *L. reuteri* supernatants, secrete factors that enhance osterix (transcription factor involved in osteoblast differentiation) expression in MC3T3-E1 osteoblasts. Together, these data suggest that *L. reuteri* secreted factors regulate T-lymphocytes which play an important role in mediating the beneficial effects of *L. reuteri* on bone density.

## Introduction

Osteoporosis is a growing medical and socioeconomic issue world-wide. Patients with osteoporosis exhibit systemic low bone mineral density and deteriorated bone microarchitecture and therefore are at an increased risk of fracture^1,2^. Both women (1 in 2) and men (1 in 4) older than 50 years will experience an osteoporotic fracture in their lifetime, with treatment costs in the US alone expected to rise over $25 billion annually by 2025. While numerous therapies are available for reducing bone loss, they are associated with side-effects or high costs^3,4^. Therefore, some patients choose to either not start the course of treatment or do not see it through to conclusion, increasing their risk of having an osteoporotic fracture and further complications^5^. For these reasons novel osteoporosis therapeutics that are low-cost and that have fewer side-effects are desired.

In recent years the gut-bone axis has gained significant attention. In this regard, intestinal microbiota dysbiosis is associated with the pathogenesis of diseases including inflammatory bowel disease (IBD), diabetes, obesity and rheumatoid arthritis all of which are associated with bone loss and the development of secondary osteoporosis^6–10^. In contrast, probiotic bacteria supplementation has been demonstrated to be beneficial to bone health^11–13^. Our lab has previously shown that administration of *Lactobacillus reuteri* (*L. reuteri*) 6475 for 4 weeks to male but not female mice increased femoral trabecular bone density^14^. The beneficial bone effect of *L. reuteri* was observed in female mice only under a mild inflammatory state or if they were ovariectomized, suggesting a role for inflammation and/or sex hormones in modulating probiotic activity^11,15^. Probiotic bacteria have also been tested for their potential therapeutic effects in a number of diseases associated with adverse bone loss^16,17^. Our lab has demonstrated that *L. reuteri* 6475 prevents bone loss associated with type 1 diabetes, low estrogen, as well as dysbiosis-induced bone loss in mice^11,18,19^. Still, the exact mechanism by which *L. reuteri* 6475 in the intestinal tract exerts a systemic effect to promote bone health remains to be fully elucidated.

The current paradigm for interaction between bacteria and the immune system in the intestine involves the uptake of bacteria by microfold cells (M cells) in the follicle-associated epithelium of Peyer’s patches, transfer of bacteria via channels in goblet cells, or paracellular or transcytotic transport across the epithelium^20^. These bacteria are then taken up by antigen presenting cells (APCs) in the Peyer’s patches, lamina propria or mesenteric lymph nodes (MLNs) which then activate T-cells^20^. Given that *L. reuteri* is administered orally, we wanted to examine whether the immune system, specifically the lymphocytes are involved in the beneficial effects of this probiotic bacteria on bone health. Using Rag knockout mice, deficient in mature T- and B- lymphocytes, we demonstrate that the benefits of *L. reuteri* on bone health require lymphocytes. In *ex vivo* studies, we demonstrate that live *L. reuteri* and its secreted components stimulate T-lymphocytes to further secrete factors that can benefit osteoblasts. Together, our studies provide potential host as well as bacterial mechanisms by which *L. reuteri* enhances bone density in male mice.

## Materials and Methods

### Ethical approval

Animal protocols were approved by the Michigan State University Institutional Animal Care and Use Committee and conformed to NIH guidelines.

### Animals and experimental design

For all experiments, mice were obtained from Jackson Laboratories (Bar Harbor, ME) and housed at Michigan State University animal facility (specific pathogen free). Mice were maintained in a light-dark cycle (12:12-h) at 23 °C (5 animals per cage) for the duration of the studies. Shipped mice were allowed to acclimate for at least one week before experiments were conducted.

For *in vivo* experiments, male mice (12 weeks of age) wild-type (C57BL/6) and Rag knockout (Rag1^tm1M^°^m^, C57BL/6 background) were divided into four cohorts randomly as follows: WT (+/− LR) and KO (+/− LR). Both strains of mice were housed in the same room and on the same rack to ensure adaptation to identical housing environment and to prevent cage effect. *L. reuteri* treated mice received 3.3 × 10^8^ cfu/ml of *L. reuteri* in drinking water for four weeks. Water bottles were changed every other day, and fresh *L. reuteri* added. Control mice received just water. Mice had access of food (Teklad 7914 chow, Madison, WI) and water *ad libitum*. At the experimental endpoint mice were sacrificed by overdose of isoflurane anesthesia followed by cervical dislocation.

### Bacterial culture conditions

*L. reuteri* ATCC PTA 6475 was cultured as previously reported^14,15,19^. Breifly, *L. reuteri* was streaked on deMan, Rogosa, Sharpe media (MRS, Difco) - agar plates and incubated under anaerobic conditions at 37 °C, for a maximum of 1 week. For live bacteria, multiple colonies were selected and anaerobically cultured in 10 ml of MRS broth (16–18 h @ 37 °C). Bacteria were washed with sterile PBS (2 × ) by centrifugation (10 minutes @ 4000 RCF) to remove all traces of MRS broth. Following the final wash, bacteria were re-suspended in 10 ml RPMI (no serum or antibiotics). Bacterial concentration was calculated using the OD_600_ value. For heat killed bacteria, 1 ml of bacterial suspension was heated to 70 °C for 50 minutes. Bacteria viability was confirmed by culturing anaerobically overnight at 37 °C on MRS plates.

For *in vivo* experiments *L. reuteri* was cultured as above with a few modifications to produce a higher concentration of bacteria. After sub-culturing into fresh MRS broth (10 ml) for 16–18 hours, the overnight culture was further incubated in additional fresh MRS broth (800 ml) until log phase (OD_600_ = 0.4). *L. reuteri* was pelleted by centrifugation (4000 RCF for 10 minutes) and washed 3x with PBS. The final pellet was re-suspended in sterile PBS (60 ml) and one milliliter (ml) aliquots were made and stored at −80 °C until further use. Colony-forming units per milliliter (cfu/ml) were calculated the day before treatment by plating 10 µl of the aliquots onto MRS agar plates overnight at 37 °C. Mice were administered *L. reuteri* (3.3 × 10^8^ cfu/ml) *reuteri* in the drinking water. Drinking water was refilled with fresh water and/or probiotic 3 × /week.

### Generation of bacterial conditioned media

Bacterial conditioned media was generated as described before^18^. Briefly, after culture (as described above), bacteria was resuspended in RPMI and incubated for 3 hours (@ 37 C) anaerobically on a rocker. The culture was then subjected to centrifugation (4000 RCF for 10 min) to pellet the bacteria. The conditioned RPMI media (supernatant) was collected, pH determined and sterile filtered (0.22 µm). Control media was generated by adjusting the pH of RPMI media to that of the bacteria CM using lactic acid (Sigma, St Louis, MO, USA). Control media and CM were aliquoted and stored at −80 °C until further use.

### Generation of <3 kDa Fraction of *L. reuteri* Conditioned Media

*L. reuteri* conditioned media or RPMI control media was added to an Amicon Ultra-15 3 K centrifugal filter unit (EMD Millipore, Billerica, MA, USA). The filter unit was then centrifuged for 30–60 min (4000 RCF). Flow through containing the <3 kDa fraction was collected, sterile filtered (using 0.22 µm filter), aliquoted and then stored at −80 °C until further use.

### Solid-Phase extraction (SPE) fractionation of conditioned media

Fractionation of *L. reuteri* conditioned media or RPMI control media based on solubility was performed using Oasis PRiME HLB extraction columns (6 cc, 500 mg from Waters, Milford, MA, USA). Briefly, 5.0 mL of CM or RPMI was added to the column and flow through was collected (load). Components retained in the column were collected by washing with 5.0 mL dH_2_0 (wash) followed by elution with 5.0 mL of 90% acetonitrile/10% dH_2_0 v/v (elute). Solvents were removed until dryness from each of the load, wash, and elute fractions by evaporation under vacuum at room temperature using a SpeedVac. Samples were re-dissolved in a comparable volume of RPMI, aliquoted and then stored at −80 °C until further use.

### Carboxyfluorescein succinimidyl ester (cfse) staining of bacteria and analysis of translocation to the mesenteric lymph nodes

Live *L. reuteri*, generated as described, was stained with CellTrace CFSE cell proliferation kit (ThermoFisher Scientific, USA) according to manufacturer’s protocol (ThermoFisher Scientific, USA). Male mice (C57BL/6, 14 weeks old), were gavaged with CFSE-stained *L. reuteri (*300 µl of 1 × 10^9^ cfu/ml). At various time points after gavage, animals were sacrificed and mesenteric lymph nodes isolated^21^, homogenized and presence of CFSE^+^ bacteria analyzed by flow cytometry.

### Mesenteric lymph node stimulation

Mesenteric lymph nodes (MLNs) were isolated, homogenized and plated at 1 × 10^5^ cells per well in RPMI media (96-well plate). For CD3^+^ MLN cultures, CD3^+^ cells were isolated using magnetic cell sorting (Miltenyi Biotec, San Diego, CA, USA) and plated at 1 × 10^5^ cells per well. Cells were then cultured in the presence or absence of live or heat killed (HK) *L. reuteri* 6475, at 1, 10 and 100 MOI (multiplicity of infection) for 4 days (37 °C 5% CO_2_). Cells were then collected and subjected to flow cytometric analysis for the respective cytokines.

### Cell isolation from spleen

Spleens were homogenized and re-suspended in RPMI media as described before^22^. For isolation of naïve CD4^+^ T cells, magnetic Naïve CD4^+^ T Cell Isolation Kit (Miltenyi Biotec) was used. Isolated cells were cultured (@1 × 10^5^) under non-polarising conditions, treated with either conditioned media (whole or fractionated) or live or heat killed (HK) *L. reuteri* 6475 at the indicated MOI for 4 days_._ Cells were then subjected to flow cytometric analysis. In some experiments cells were also stimulated with CD3/CD28 antibodies (CD3: 10 µg/ml; 145-2C11; CD28 (5 µg/ml; 37.51, BD Biosciences) for 4 days and then subjected to flow cytometry.

### Flow cytometry analysis

For analysis, cells were pelleted and supernatant removed. Flow cytometry staining was performed as previously reported^15,23,24^. Briefly, cells were incubated with Fc block (BD Pharmingen, CA, USA) for 15 min before being stained with anti-mouse CD3-APC AlexaFluor 780 (500A2, eBioscience) and anti-mouse CD4-eF450 (RM 4–5, eBioscience) for 30 minutes at 4 °C. Cells were washed 3X in assay buffer (PBS, 0.5% bovine serum albumin (BSA), 5 mM EDTA) followed by permeabilization using cytofix/cytoperm (BD Pharmingen)^23^. Intracellular staining for cytokines was performed with anti-mouse IL-10 FITC (JES5-16E3, eBioscience), anti-mouse IL-17A PE (TC11-18H10, BD Bioscience), anti-mouse IFNγ APC (XMG1.2, eBioscience) and anti-mouse LAP (TGFβ) PerCP-eF710 (TW7-16B4, eBioscience). Data were acquired on a BD LSRII (Becton Dickinson, Franklin Lakes, NJ) and analyzed with FlowJo (Version 10; FlowJo, LLC, Ashland OR)^15^.

### *In vitro* cell culture system

Preosteoblast MC3T3-E1 cells (CRL-2593; ATCC, Manassas, VA) were cultured as described previously^25^. Briefly, cells were cultured in complete alpha minimal essential media (α-MEM) containing 10% fetal bovine serum (FBS) (Invitrogen and Atlanta Biologicals, Atlanta, GA) and 1% Penicillin-Streptomycin (Life Technologies). Passages between 18 and 24 were used for the experiments. For gene expression analysis, cells were plated in 96-well plates (@ 20,000 cells/well) (Corning Incorporated, Corning NY). Following 24 hours of plating in complete α-MEM, cells were treated as indicated for 6 hours. RNA extraction was completed as described below.

For assessment of intracellular ATP, MC3T3-E1 cells were plated @10,000 cells/well in 96-well plates (white-walled from Corning Incorporated, Corning NY). Twenty four hours after plating, cells were treated as indicated for 6 hours. Intracellular ATP levels were assessed using the ApoSensor Cell Viability Assay kit (BioVision, San Francisco, CA) and Luminescence was measured by Synergy/neo2 multi-mode plate reader and calculated with Gen5 software (Bio-Tek). Each experiment was done in duplicates.

### RNA extraction

RNA extraction was performed using TriReagent (Molecular Research Center, Cincinnati, OH) and RNA integrity was verified using agarose-gel electrophoresis^15,26,27^. Complementary DNA was synthesized by reverse transcription using Superscript II Reverse Transcriptase Kit and oligo dT primers (Invitrogen, Carlsbad, CA)^28^. Complementary DNA was amplified by PCR using iQ SYBR Green supermix (Bio-Rad Laboratories, Hercules, CA). Real time PCR was carried out for 40 cycles (95 °C for 15 seconds, 60 °C for 30 seconds, and 72° C for 30 seconds) using an iCycler thermal cycler and data evaluated using the iCycler software. Reactions without cDNA were used as negative controls. RNA levels of hypoxanthine guanine phosphoribosyl transferase (HPRT) did not change with treatment and therefore HPRT was used as internal control^19^. The following primers used for real-time PCR: *HPRT* (Forward, **5**′-AAGCCTAAGATGAGCGCAAG-**3**′, Reverse, **5**′-TTACTAGGCAGATGGCCACA), *Osterix* (Forward **5**′-CTGCGGAAAGGAGGCACAAAGAAG-**3**′, Reverse, **5**′-GGGTTAAGGGGAGCAAAGTCAGAT-3′), *Bax* (Forward **5**′ GACAGGGGGCTTTTTGCTA **3**′, Reverse, **5**′-TGTCCACGTCAGCAATCATC-**3**′), *Bcl-2* (Forward **5**′-GACAGAAGATCATGCCGTCC-**3**′, Reverse, **5**′-GGTACCAATGGCACTTCAAG-**3**′).

### Microcomputed tomography (μCT) bone imaging

Microcomputed tomography was performed as previously described^15^. After euthanasia, femoral bones were collected and fixed in 10% formalin for 24 hours. Bones were then transferred to 70% ethanol and scanned using a GE Explore Locus microcomputed tomography (μCT) system at a voxel resolution of 20 μm obtained from 720 views^28^. For each run bone from all groups were included. In addition, a calibration phantom was included to standardize gray scale values and maintain consistency. To separate bone from bone marrow a fixed threshold (841) was used. Femur trabecular bone analyses was performed from 1% of the total length proximal to the growth plate, extending 2 mm toward the diaphysis, and excluding the outer cortical bone. Trabecular bone volume fraction (BVF), bone mineral content (BMC), bone mineral density (BMD), thickness (Tb. Th), spacing (Tb. Sp), and number (Tb. N) were computed using GE Healthcare MicroView software. Femoral trabecular isosurface images were taken from a region in the femur where analyses were performed measuring 1.0 mm in length and 1.0 mm in diameter. Cortical measurements were performed in a 2 × 2 × 2 mm cube centered midway down the length of the bone.

### DNA extraction from colonic and fecal samples, 16S rRNA gene amplification, and sequencing

DNA was extracted from colonic and fecal samples as previously described^19^. Briefly, fecal samples were homogenized in a BioSpec Mini-Beadbeater in the presence of buffer ATL (Qiagen). Following homogenization, Proteinase K (Qiagen) was added and samples were incubated for 30 minutes at 55 °C. Samples were homogenized again for another minute and incubated for additional 30 minutes at 55 C. DNA extraction was performed using Qiagen DNeasy Blood and Tissue kits as described previously^11,29^. Bacterial 16S sequences spanning variable region V4 were amplified by PCR with primers F515/R806 with a dual indexing approach. Sequencing was done using Illumina MiSeq.^30^. PCR reactions (20 µl) containing DNA template (40 ng), 1X Phusion High-Fidelity Buffer (New England Biolabs), dNTPs (200 μM from Promega or Invitrogen), primers (10 nM), Phusion DNA Polymerase (0.2 units from New England Biolabs), and PCR grade water were performed in an Eppendorf Prothermal cycler. The initial denaturation was performed at 98 °C for 30 s, followed by 30 cycles of 10 s at 98 °C, 20 s at 51 °C, and 1 min at 72 °C. Replicates samples were pooled and purified with Agencourt AMPure XP magnetic beads (Beckman Coulter). Samples were quantified using the QuantIt High Sensitivity DNA assay kit (Invitrogen) and pooled at equimolar ratios. The quality of the DNA samples was evaluated with the Bioanalyzer High Sensitivity DNA Kit (Agilent).

### Microbial community analysis

Sequence data were processed using the MiSeq pipeline for mothur using software version 1.38.1^31^ as described previously^19,29^. Sequences were clustered into operational taxonomic units (OTUs) with 97% similarity using the average-neighbor algorithm in mothur. A total of 871 OTUs were identified across all samples with an average rarefaction depth of 54,791 reads per sample. Diversity analyses (alpha and beta) and visualization of microbiome communities were performed with R, utilizing the phyloseq package^32,33^. The Bray-Curtis dissimilarity matrix was used to describe differences in microbial community structure.

### Statistical analysis

All measurements are presented as the mean ± SEM or as box plots (whiskers indicate minimum to maximum values). Significance was tested using either Student’s t test (2 groups) or ANOVA (more than 2 groups) with Bonferroni’s multiple comparison test for the post-hoc test. Statistical analysis was performed using GraphPad Prism software version 7 (GraphPad, San Diego, CA, USA). Significant outliers (if present and indicated in figure legend) were removed using the ROUT test for outliers. A p-value of < 0.05 was considered significant.

## Results

### *L. reuteri* requires lymphocytes to Exert Beneficial Effects on Bone

**Figure 1:**
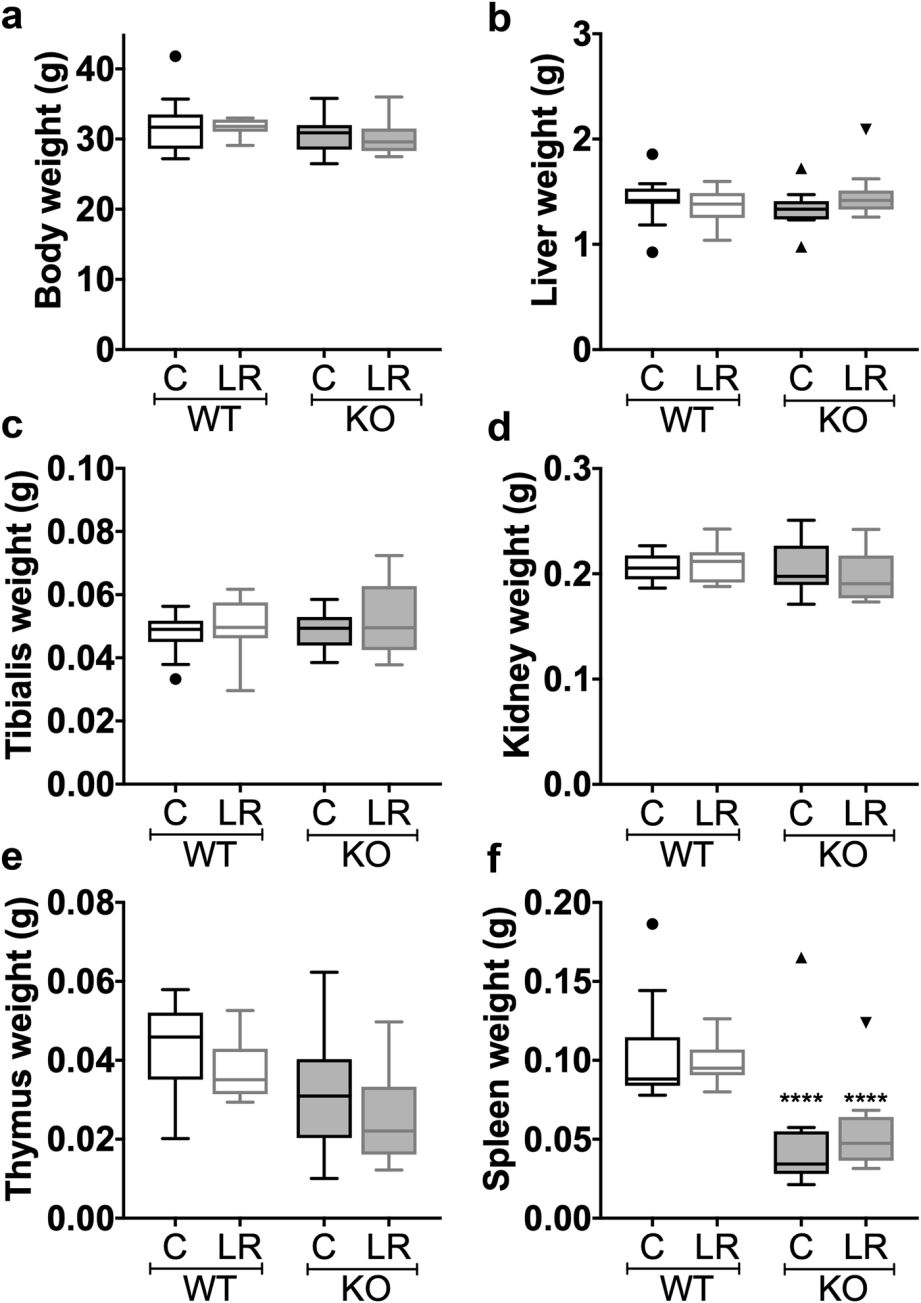
General body parameters. 12 weeks old C57BL/6 and Rag KO male mice were supplemented with water (n = 11) or *L. reuteri* (n = 10) for 4 weeks. General body parameters were measured the day of harvest. (**a**) body, (**b**) liver, (**c**) tibialis, (**d**) kidney, (**e**) thymus, and (**f**) spleen weight in grams (**g**). *L. reuteri* treatment had no effect on general body parameters. Data presented as box plots with Whiskers using Tukey method. Statistical analysis performed by One-way ANOVA with Bonferroni’s multiple comparison test. ****p < 0.0001.

**Figure 2:**
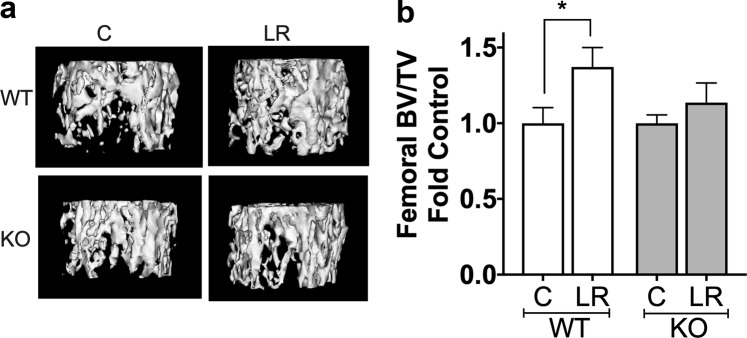
*L. reuteri* requires lymphocytes to exert beneficial effect on Bone. Twelve week old C57BL/6 and Rag KO male mice were supplemented with water (n = 11) or *L. reuteri* (n = 10) for 4 weeks. Femoral bone was collected, and trabecular and cortical bone analyzed by µCT. (**a**) representative micro-computed tomography isosurface images by uCT. (**b**) bone volume fraction (BV/TV) quantitative data obtained from the distal femur trabecular bone of control and *L. reuteri* treated mice. Statistical analysis performed one-way ANOVA with Bonferroni’s multiple comparison test. *p < 0.05.

Previous studies have revealed that supplementation with the probiotic *L. reuteri* ATCC 6475 can have a beneficial effect on bone health^11,14,15,19,34^ though the exact mechanisms are currently unknown. We and others have shown that *L. reuteri* has immuno-regulatory properties *in vitro* and *in vivo*, suggesting a role for the immune system in modulating the effects of *L. reuteri* on bone density^11,14,35^. Although previous studies have shown that *L. reuteri* can modulate the immune system, the specific role of different immune cells in mediating effects on bone are not well known. To address this, we utilized male mice that are deficient in mature T and B lymphocytes (Rag1 knockout; KO) and wild type (WT) controls. WT and KO mice were administered *L. reuteri* in their drinking water for 4 weeks. Control mice received water without the probiotic. After 4 weeks, *L. reuteri* administration had no significant effect on body weight or weights of organs including liver, muscle (tibialis), kidney, thymus and spleen (Fig. 1). As expected, a significant decrease in thymus and spleen weights was observed in the KO group compared to the WT mice (Fig. 1e,f). As we reported earlier^14^, oral *L. reuteri* treatment significantly increased trabecular femoral bone density by 37% in the WT mice (Fig. 2, p < 0.05). In contrast, *L. reuteri* treatment did not enhance trabecular femoral bone density in the KO mice (Fig. 2), suggesting that *L. reuteri* requires lymphocytes to increase trabecular bone density. Consistent with our findings, bone mineral density (BMD, p < 0.05) and trabecular thickness increased (Tb. Th, p < 0.05), while trabecular spacing decreased (Tb. Sp, p < 0.05) in the *L. reuteri* treated WT mice (Table 1). Analysis of the cortical regions of the femur (diaphyseal region) showed that marrow area was decreased and outer perimeter increased by *L. reuteri* in the WT mice but not in the KO. Other parameters did not demonstrate any significant differences between the groups (Table 1).

**Table 1:** Femoral Bone Parameters.

Parameter:	Wild Type		Rag knockout	
	C (n = 11)	LR (n = 10)	C (n = 11)	LR (n = 10)
**Femur trabecular**				
BV/TV	32.28 ± 3.33	44.45 ± 4.16^#^	40.53 ± 2.23	46.05 ± 5.25
BMD (mg/mL)	243.5 ± 8.13	282.5 ± 12.41^#^	265.2 ± 6.27	290.3 ± 18.44
BMC (mg)	0.46 ± 0.02	0.52 ± 0.01	0.51 ± 0.01	0.52 ± 0.02
Tb. Th. (mm)	0.04 ± 0.002	0.06 ± 0.004^#^	0.05 ± 0.002	0.07 ± 0.006*
Tb. N.(1/mm)	6.94 ± 0.46	7.24 ± 0.24	7.23 ± 0.23	6.47 ± 0.35
Tb. Sp. (mm)	0.10 ± 0.01	0.08 ± 0.008	0.08 ± 0.006	0.07 ± 0.007
**Femur Cortical**				
Cortical area (mm^2)	0.96 ± 0.05	0.93 ± 0.04	0.94 ± 0.04	0.93 ± 0.04
Marrow area (mm^2)	0.81 ± 0.04	0.68 ± 0.03^#^	0.67 ± 0.02	0.66 ± 0.04
Mean thickness (mm) Inner	0.24 ± 0.008	0.25 ± 0.006	0.25 ± 0.007	0.25 ± 0.008
Inner perimeter (mm)	3.76 ± 0.07	3.73 ± 0.04	3.62 ± 0.09	3.77 ± 0.13
Outer perimeter (mm)	6.77 ± 0.43	7.96 ± 0.26^#^	7.24 ± 0.42	8.08 ± 0.44
BMD (mg/mL)	784.3 ± 13.43	779.7 ± 7.17	784.1 ± 7.14	800 ± 11.65
BMC (mg)	0.01 ± 0.001	0.02 ± 0.00	0.01 ± 0.00	0.02 ± 0.00

### Effect of *L. reuteri* on α and β diversity in gut microbiota in male mice

**Figure 3:**
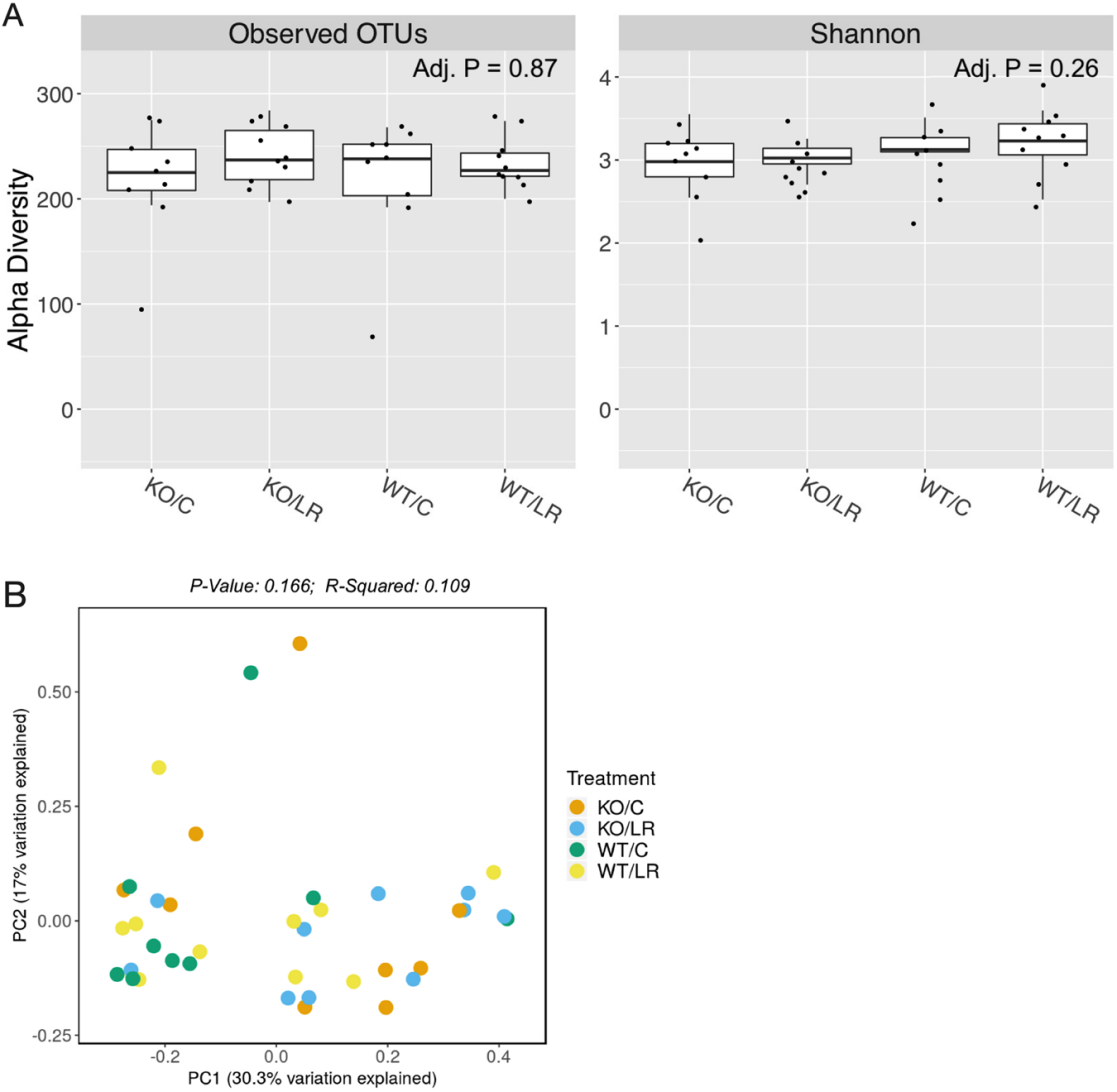
*L. reuteri* effects on gut microbiota. Twelve week old C57BL/6 and Rag KO male mice were supplemented with water or *L. reuteri* for 4 weeks as indicated in Fig. 2. Fecal samples were collected at the day of harvest and analyzed by 16S rRNA sequencing as described in the methods. (**A**) Plots of alpha-diversity metrics of observed operational taxonomic units (OTUs) (richness) and Shannon diversity (richness and evenness) of the different treatment groups. (**B**) Principle coordinate analysis of beta-diversity based on Bray-Curtis (richness and evenness) of the different treatment groups. WT/C = wild-type control, WT/LR = wild-type *L. reuteri* treated, KO/C = Rag1 KO control, KO/LR = Rag1 KO *L. reuteri* treated. N = 9–10/group.

To assess if probiotic administration significantly modulated intestinal microbiota diversity in male mice and whether a difference in microbiota diversity could account for the lack of effect of *L. reuteri* in the Rag KO mice, we extracted DNA from colonic microbiota and performed 16S rRNA sequencing. Diversity metrics that utilize species richness and evenness (Bray-Curtis) showed no significant separation between the groups (WT±LR and KO±LR) (Fig. 3). Also, no significant differences were found in α diversity both in terms of OTUs and Shannon index (Fig. 3). These data suggest that *L. reuteri* treatment in either WT or Rag KO mice does not cause broad changes in bacterial communities in male mice. This result does not rule out changes in microbiota function or changes in specific bacterial species that may be impacted by *L. reuteri* supplementation. Given the absence of mature lymphocytes in Rag KO mice, we reasoned that the lymphocytes likely play an important and direct role in *L. reuteri* effects on bone.

### Effect of live *L. reuteri* on cytokine expression in Whole MLN *Ex Vivo* Cultures

**Figure 4:**
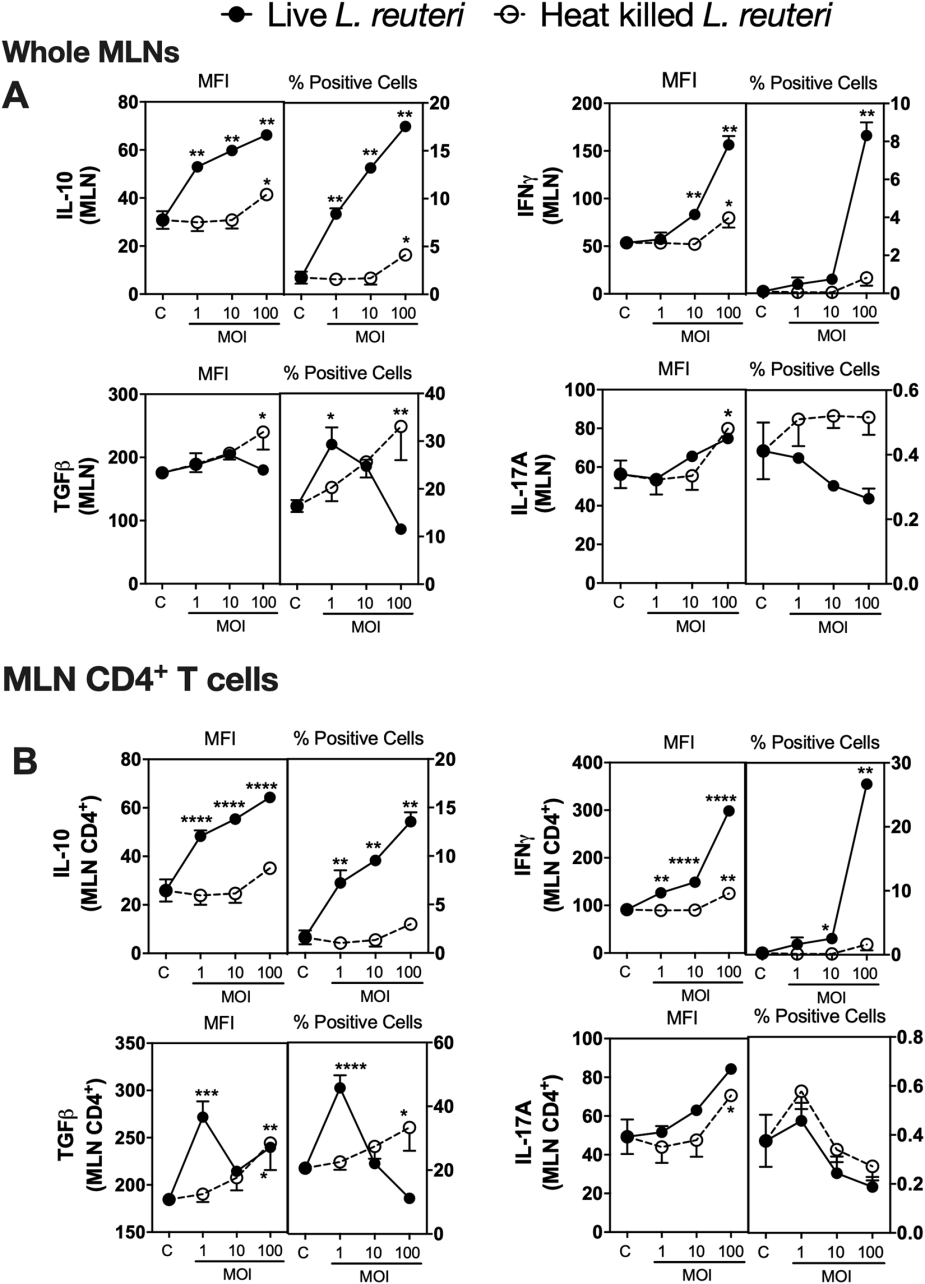
Effect of live and heat killed *L. reuteri* 6475 on cytokine expression in whole MLN cultures. Mesenteric lymph nodes were isolated from male C57BL/6 mice (12–18 weeks), homogenized and cultured with live or heat killed *L. reuteri* 6475 at an MOI of 1, 10 or 100 for 4 days. Cells were analyzed using flow cytometry for expression of IL-10, INFγ, IL-17 A and TGFβ. Results for MFI and % positive cells were analysed as (**A**) the whole MLN or (**B**) gated on the MLN CD4^+^ T cells. Statistical analysis performed by 2-way ANOVA with Bonferroni’s multiple comparison test. *p < 0.05, **p < 0.01, ***p < 0.001, ****p < 0.0001 compared to control. n = 5.

Because our *in vivo* studies were performed with *L. reuteri* administered orally, we reasoned that lymphocytes in MLNs would be one of the sites of *L. reuteri* or its products could interact with lymphocytes. Consistent with our reasoning other studies have shown that probiotics including *L. reuteri* can translocate to the MLNs following oral administration^36^. Using a CFSE (5(6)-Carboxyfluorescein N-hydroxysuccinimidyl ester) labelled *L. reuteri* and flow cytometry analysis, we confirmed that following 6 hours after oral gavage, *L. reuteri* can be detected in the MLNs (data not shown). To understand the effect of *L. reuteri* and its secreted products on lymphocytes in MLNs, we treated MLNs *ex vivo* with *L. reuteri* for 4 days and assessed expression of cytokines that have been shown to regulate bone health. We focused on IL-10 and TGFβ (can enhance bone health)^37–41^ as well as IFNγ (negatively influences bone health) and IL-17A (has both negative and positive effects on bone)^42–44^. These cytokines were assessed using flow cytometry. Treatment of MLN cultures with live *L. reuteri* significantly increased expression (as indicated by median fluorescent intensity; MFI) of IL-10 and IFNγ in a concentration-dependent manner (*p* < 0.01) (Fig. 4). In addition, the number of IL-10 + and IFNγ + cells also increased significantly (*p* < 0.01). In contrast, TGFβ expression showed minimal change with live *L. reuteri*. However, TGFβ + cell numbers were differentially modulated at different MOI, with an MOI of 1 causing an increase (*p* < 0.05), and higher MOIs showing no difference. While expression of IL-17A increased only at the highest MOI, IL-17A^+^ cell numbers were not affected by live *L. reuteri* (Fig. 4A). Gating within the CD4^+^ T cell population also revealed similar expression and + cell profiles, except for TGFβ which showed a biphasic response with both MFI for TGFβ as well as TGFβ + cell numbers (Fig. 4B). Compared to live *L. reuteri*, effect of heat killed *L. reuteri* was not consistent and in some cases showed a modest response only at high concentrations (*p* < 0.05) (Fig. 4B). Together, these results demonstrate that live but not heat killed *L. reuteri* can significantly induce concentration-dependent effects on the MLN immune cell cytokine profiles and the majority of these effects are similar between the whole MLN and the MLN CD4^+^ T cell population.

**Figure 5:**
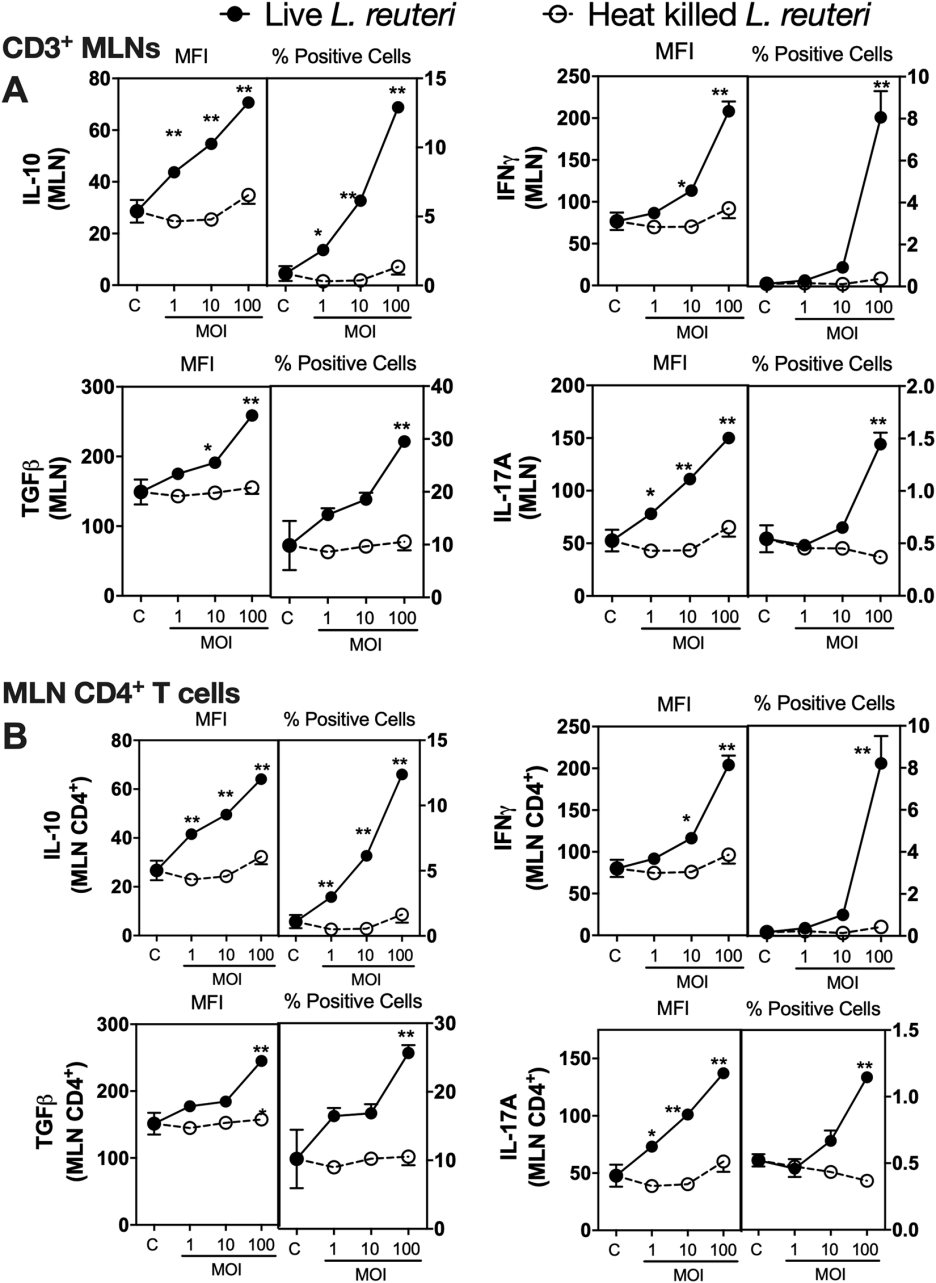
Effect of *L. reuteri* 6475 on cytokine expression in CD3^+^ lymphocytes isolated from MLNs. Mesenteric lymph nodes were isolated from male C57BL/6 mice (12–18 weeks), homogenized and CD3^+^ cells obtained by magnetic separation. CD3^+^ cells were cultured with live or heat killed *L. reuteri* 6475 at an MOI of 1, 10 or 100 for 4 days. Cells were analyzed using flow cytometry for expression of IL-10, INFγ, IL-17 A and TGFβ. Results for MFI and % positive cells were analysed as (**A**) CD3 + cells or (**B**) gated on the MLN CD4^+^ T cells. Statistical analysis performed by 2-way ANOVA with Bonferroni’s multiple comparison test. *p < 0.05, **p < 0.01 compared to relevant control. n = 5.

Mesenteric lymph nodes contain a multitude of different cells types including antigen presenting cells (APCs). To identify whether the effects of *L. reuteri* on the CD4^+^ T cells was indirect via APCs or direct on the T cells, CD3^+^ cells were isolated from whole MLNs and cultured with either live or heat killed *L. reuteri*. As with the whole MLN cultures, addition of live *L. reuteri* to CD3^+^ MLN cells had a profound effect on cytokine expression (Fig. 5A). Live *L. reuteri* significantly increased expression of IL-10, IL-17A, TGFβ and IFNγ in a concentration-dependent manner as determined by flow cytometry. Similarly, number of CD3^+^ cells expressing these respective cytokines was also significantly increased by live *L. reuteri* treatment. Compared to this, heat killed *L. reuteri* had no significant effect on expression of any of the cytokines examined. When gated within the CD4+ T-cells, the results were similar to that of CD3+ T cells (Fig. 5A).

**Figure 6:**
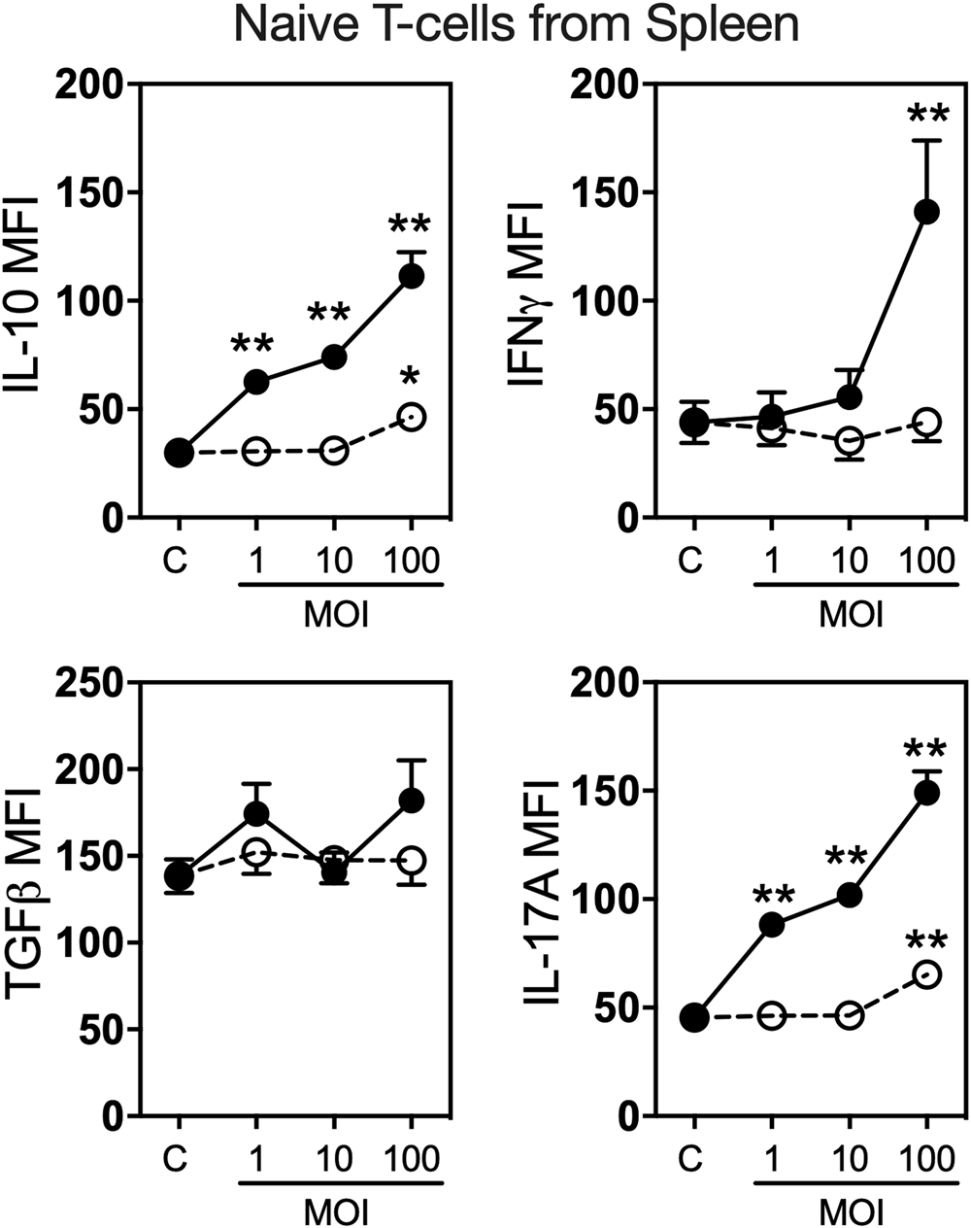
Effect of *L. reuteri* on naïve splenic CD4^+^ T Cells cytokine expression. Spleens were isolated from male C57BL/6 mice (12–18 weeks) and naïve CD4^+^ T cells obtained by magnetic separation. Cells were cultured under non-polarising conditions with (**a**) live or heat killed *L. reuteri* 6475 at an MOI of 1, 10 or 100 for 4 days and cytokine expression (IL-10, INFγ, IL17A and TGFβ) analyzed by flow cytometry. Statistical analysis performed by 2-way ANOVA with Bonferroni’s multiple comparison test. *p < 0.05, **p < 0.01 compared to control. n = 10.

Having revealed that *L. reuteri* directly modulates MLN CD4^+^ T cell cytokine expression, we next sought to determine the effects of live *L. reuteri* on naïve CD4^+^ T cells. CD4^+^ T cells were isolated from spleens by magnetic isolation and treated with either live or heat killed *L. reuteri* under non-polarising culture conditions. Comparable to the responses of isolated CD3^+^ MLN cultures, CD4^+^ splenic T cells exhibited a concentration-dependent response to live *L. reuteri* for both IL-10 and IL-17A expression (*p* < 0.01) (Fig. 6). IFNγ expression increased significantly only at an MOI of 100 (*p* < 0.01) while no effect was observed for TGFβ. Heat killed *L. reuteri* increased IL-10 (*p* < 0.05) and IL-17A (*p* < 0.01) expression modestly, only at an MOI of 100 (Fig. 6). Together, these data suggest that live *L. reuteri* is able to stimulate T-cells directly and can modulate cytokine expression in cells that have potential modulatory effects on bone health.

### Effect of *L. reuteri* secreted factors on T-lymphocyte cytokine expression

**Figure 7:**
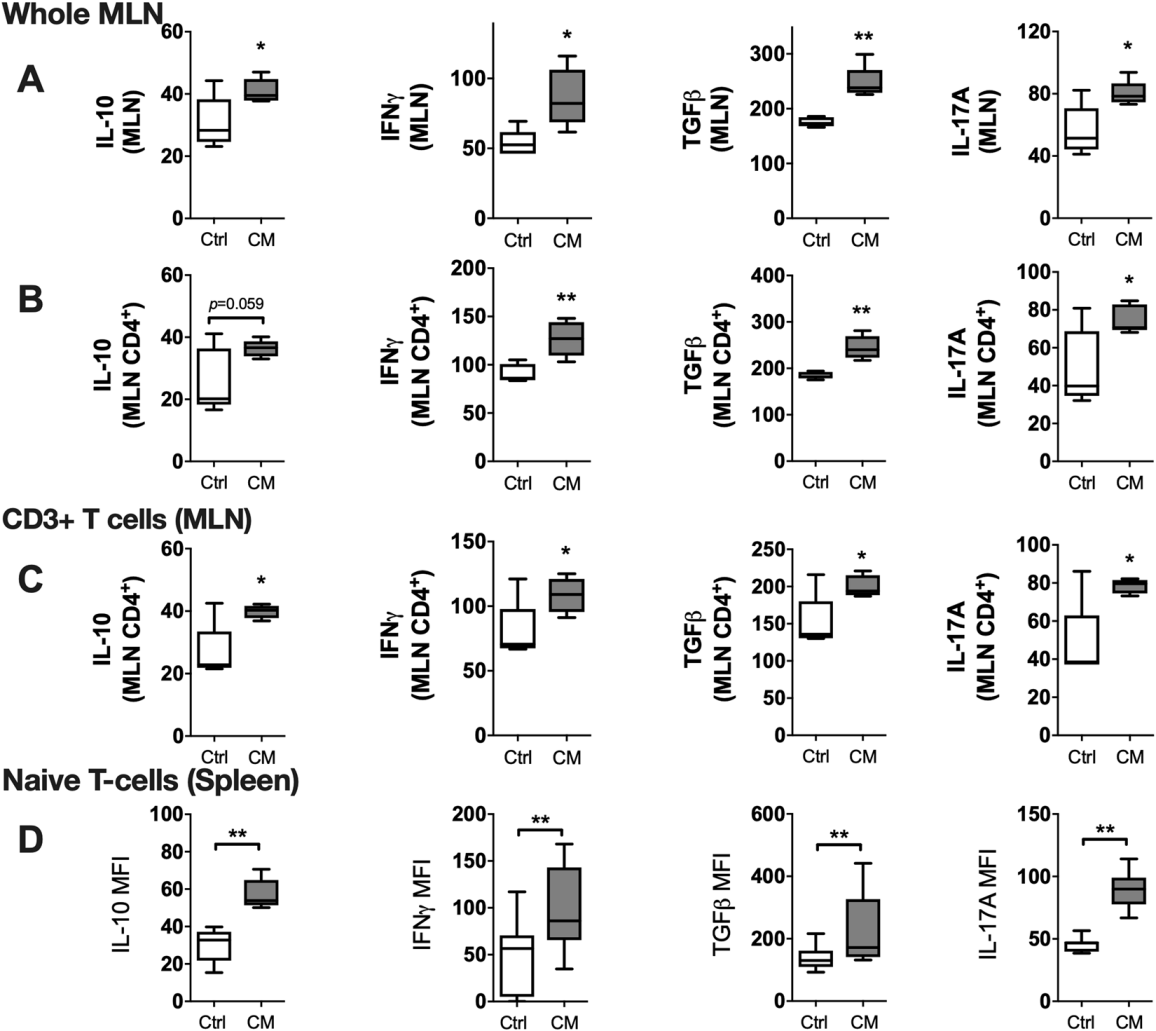
Effect of *L. reuteri* conditioned media on cytokine expression in T-cells. Mesenteric lymph nodes and spleens were isolated from male C57BL/6 mice (12–18 weeks), homogenized and cultured with *L. reuteri* 6475 CM or control media for 4 days. Expression of cytokines (IL-10, IFNγ, TGFβ and IL-17A) was assessed using Flow cytometry. (**A**) whole MLN cultures, (**B**) CD3^+^ T cells, and (**C**) naïve CD4^+^ T cells. Statistical analysis performed by unpaired t-test. *p < 0.05, **p < 0.01 compared to control. Whiskers in the box plots represent minimum to maximum values. n = 5.

To identify whether the effects of live *L. reuteri* required direct cell-cell contact or whether the effects are induced by secreted factor(s), whole MLN cultures were treated *ex vivo* with *L. reuteri* conditioned media (CM). *L. reuteri* CM significantly elevated expression of all the cytokines assessed (IL-10, IFNγ, TGFβ and IL-17A) (Fig. 7A). When gated within the CD4+ T-cells, expression of cytokines showed similar pattern (Fig. 7B). Percentage of cytokine positive cells mostly followed a similar pattern to that of the MFI (data not shown). To determine whether *L. reuteri* secreted factor(s) were responsible for the observed direct effect of *L. reuteri* on T-lymphocytes, we further treated isolated CD3^+^ T-cells from MLNs with *L. reuteri* CM (Fig. 7C). Similar to the effects on whole MLN cultures, *L. reuteri* CM treatment of isolated T-cells significantly increased CD4+ T cell expression of all four cytokines (Fig. 7C) as well as increased numbers of CD4^+^ cytokine + cells (data not shown). Similar to MLN cultures, treatment of CD4+ T cells (spleen) with *L. reuteri* CM also significantly increased the expression of all cytokines assessed (Fig. 7D). Together, these results suggest that secreted component(s) of *L. reuteri* are able to directly stimulate T-cells and significantly modulate expression of cytokines.

### RIP2 negatively regulates expression of IL-10 and IL-17A in lymphocytes

**Figure 8:**
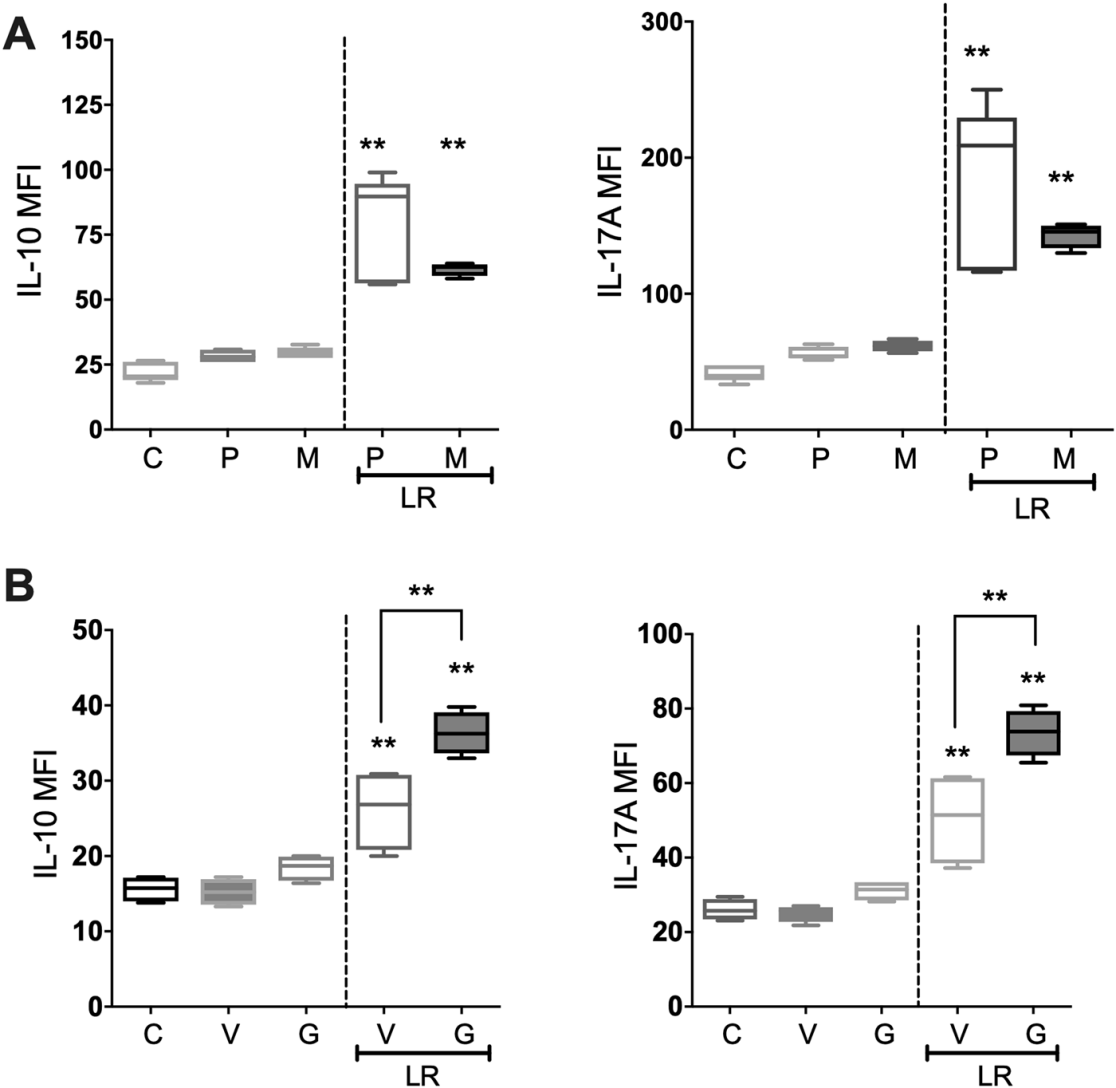
Effect of *L. reuteri* on CD4^+^ T Cells is negatively regulated by NOD pathway. Spleens were isolated from male C57BL/6 mice (12–18 weeks) and naïve CD4^+^ T cells obtained by magnetic separation. CD4^+^ T cells were pre-treated with either (**A**) MyD88 inhibitor (100 µM) or (**B**) gefitinib (20 µM) for 24 hours before culture with *L. reuteri* CM for 4 days and cytokine expression (IL-10 and IL-17A) analyzed by flow cytometry. M = MyD88 inhibitor; P = control peptide; V = DMSO vehicle control; G = Gefitinib; LR = *L. reuteri* conditioned media. Statistical analysis performed by One-way ANOVA with Bonferroni’s multiple comparison test. *p < 0.05, **p < 0.01 compared to control; ^*p* < 0.05 compared to LR treated P. n = 5.

Probiotic bacteria and *Lactobacillus* bacteria in particular have previously been shown to induce their beneficial effects through toll-like receptor (TLR) signaling, specifically TLR2^45,46^. Furthermore, bacteria are able to induce a signaling cascade through activation of the Nucleotide-binding Oligomerization Domain (NOD) pathway^47^. To determine whether *L. reuteri* secreted factors induce their modulatory effects through TLR or NOD signaling we inhibited MyD88 or RIP2 tyrosine kinase in splenic CD4^+^ T cells prior to *L. reuteri* stimulation. To determine if *L. reuteri* was acting through a TLR in a MyD88-dependent manner, we used a MyD88 peptide inhibitor (Fig. 8A). MyD88 inhibitor failed to block the effects of *L. reuteri* on IL-10 and IL-17A. To further investigate whether *L. reuteri* signaled via the NOD pathway we utilized Gefitinib, a RIP2 tyrosine kinase inhibitor which inhibits RIP2 tyrosine phosphorylation and NOD2-induced NF-κB activation and cytokine release^48^. *L. reuteri* CM significantly increased expression of IL-10, and IL-17A as expected; however, pretreatment of the cells with Gefitinib significantly augmented the expression of IL-10 (*p* < 0.01), and IL-17A (*p* < 0.01) suggesting that RIP2 negatively regulates expression of these cytokines in T-cells induced by *L. reuteri* CM (Fig. 8B).

### Fractionation of *L. reuteri* conditioned media

**Figure 9:**
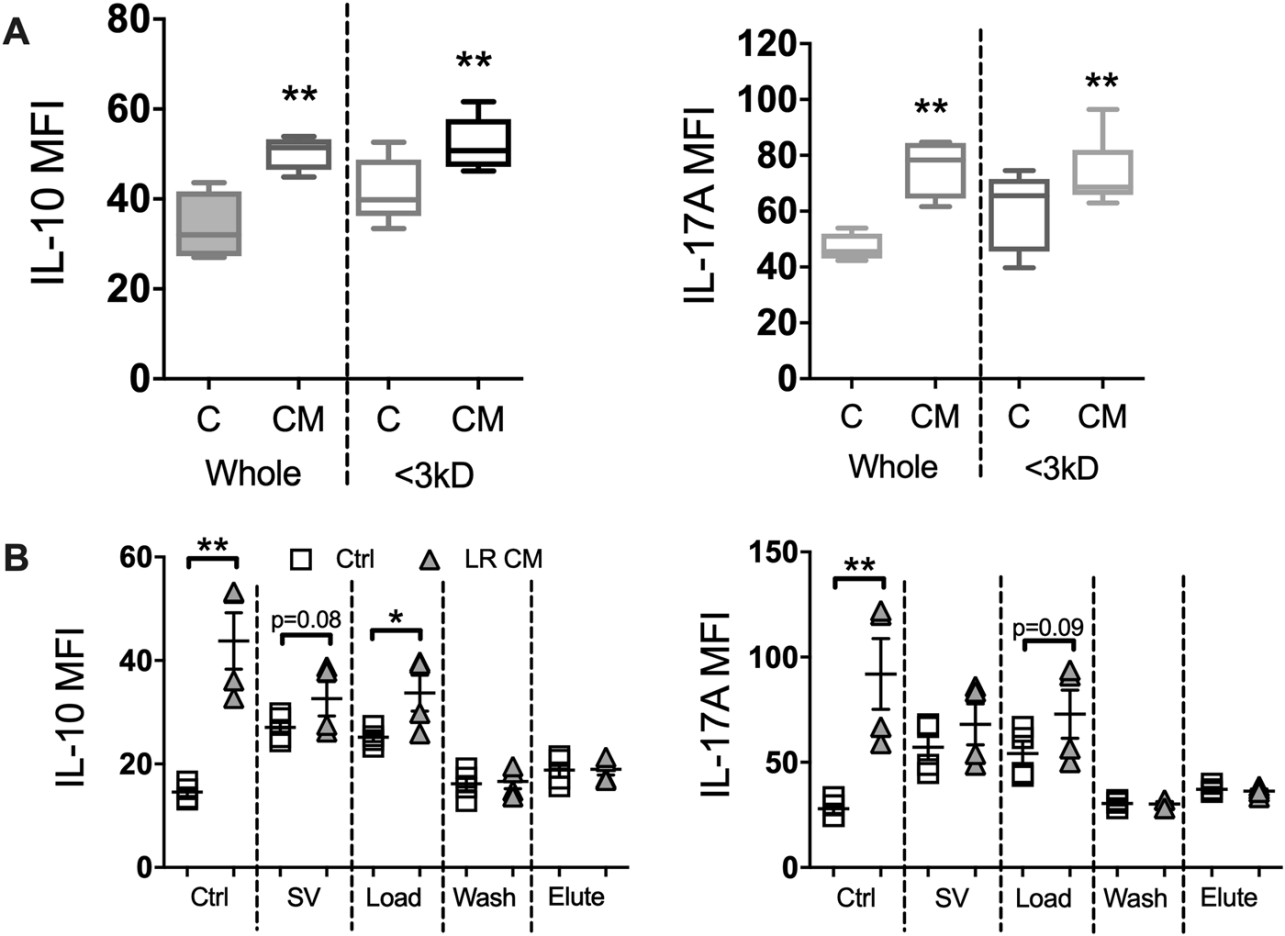
Effect of *L. reuteri* conditioned media fractions on naïve CD4^+^ T Cell cytokine expression. Spleens were isolated from male C57BL/6 mice (12–18 weeks) and naïve CD4^+^ T cells obtained by magnetic separation. CD4^+^ T cells were cultured with either a) whole or <3kD CM or b) SPE fractionated CM and expression of cytokines analyzed by flow cytometry. SV = SpeedVac. Statistical analysis performed by unpaired t-test. *p < 0.05, **p < 0.01 compared to comparable control. n = 4–5.

To identify the active components secreted by *L. reuteri* that influences T-cell cytokine expression, the conditioned media was subjected to a series of fractionations based on size and water solubility. Fractionation based on size revealed that the active component(s) in question is less than 3 kD in size (Fig. 9B). Treatment of splenic CD4^+^ T cells with the <3 kD fraction of *L. reuteri* CM significantly increased expression of IL-10 (*p* < 0.01) and IL-17A (*p* < 0.01) compared to control. To further define the active component in the *L. reuteri* CM, whole supernatants were fractionated based on water solubility using a reversed phase solid-phase extraction (SPE) column and the resulting fractions (load, wash and elute) tested for activity (Fig. 9B). Normal RPMI media and *L. reuteri* CM were also evaporated to dryness through the SpeedVac (SV) to control for any effects of the evaporation process. Analysis of cytokine expression in CD4^+^ T cells revealed significantly increased expression of IL-10 (*p* < 0.05) with the *L. reuteri* CM load fraction (unretained on the SPE column) over the control load fraction. A modest increase in IL-17A was also observed. The wash and elute fractions of the *L. reuteri* CM failed to induce any cytokine response over the control, consistent with our conclusion that the active molecule in *L. reuteri* CM is likely water soluble and therefore not retained.

### MLN and bone link

**Figure 10:**
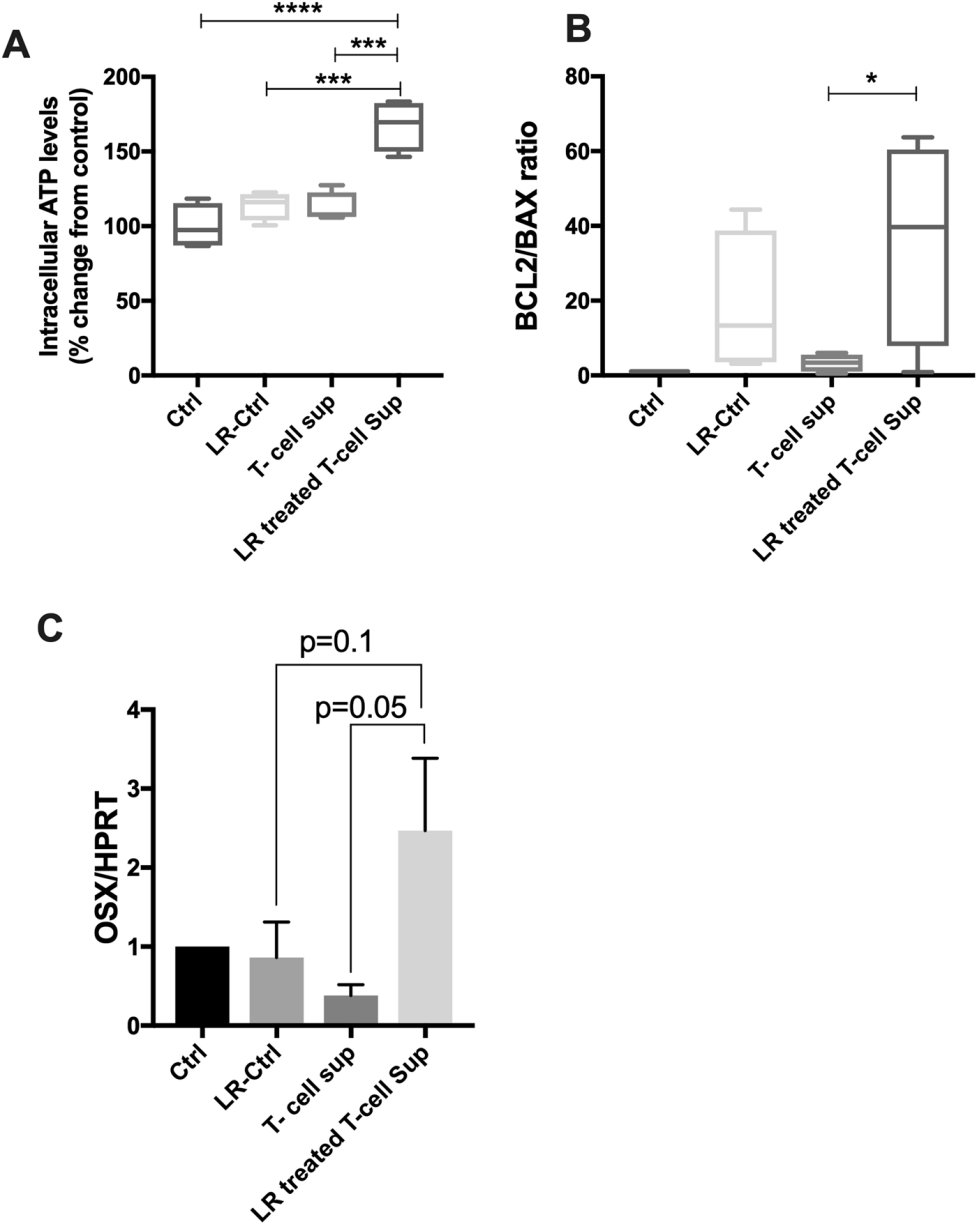
Secretory factors from *L. reuteri* CM treated T cells enhances osteoblast viability and osterix gene expression. Mesenteric lymph nodes were isolated from male C57BL/6 mice (12–18 weeks), homogenized and CD4^+^ cells obtained by magnetic separation. CD4^+^ T-cells were cultured with *L. reuteri* CM for 4 days. Supernatants from these cultures (secreted factors) were collected after 4 days and used to treat osteoblast cells for 6 hours. (**A**) cell viability was measured by intracellular ATP levels; (**B**) BCL2/BAX gene expression ratio; (**C**) osterix gene expression. Ctrl = control, LR-Ctrl = *L. reuteri* CM with no cells, T-cell sup = T cells with no treatment. Statistical analysis performed one-way ANOVA with Bonferroni’s multiple comparision test. *p < 0.05, ***p < 0.001, ****p < 0.0001. n = 5–6.

Data thus far suggest that oral *L. reuteri* administration likely interacts with MLNs and stimulates expression of multiple cytokines that have either osteoblastogenic or anti-osteoblastogenic activities. To determine the net effect of *L. reuteri* stimulation of T-cells on osteoblasts, we treated MC3T3-E1 pre-osteoblasts with T-cell supernatants (treated with or without *L. reuteri* stimulation). CD4+ T cells were isolated from MLNs and treated with *L. reuteri* CM or vehicle for 4 days. The supernatants from the T-cell cultures were then collected and added to osteoblast cultures for 6 hours. To rule out any residual activity of *L. reuteri* in the T-cell supernatant, the control *L. reuteri* CM was left in the incubator for 4 days (without any T-cells) similar to the T-cell treatment. Only the supernatants from T-cells treated with *L. reuteri* stimulated osteoblast ATP levels (Fig. 10A). Importantly, *L. reuteri* CM, incubated without any cells for 4 days did not have any activity, suggesting that the ATP inducing effect of T-cell supernatants (treated) is due to secreted factors from T-cells. Consistent with this, Bcl2/Bax ratio was also significantly enhanced by *L. reuteri* treated T-cell supernatant, suggesting that T-cell secretory factor (stimulated by *L. reuteri*) is likely able to enhance the survival of osteoblasts (Fig. 10B). Similarly, osterix expression (transcription factor important for osteoblast differentiation) was also modestly induced by the *L. reuteri* treated T-cell supernatant but not by supernatants from other groups (Fig. 10B). These data suggest that *L. reuteri* is able to stimulate T-cells in MLNs that can indeed beneficially affect osteoblasts. Taken together, these results suggest that *L. reuteri*-induced bone responses are dependent on lymphocytes and that *L. reuteri* is able to stimulate T-lymphocytes to increase expression of cytokines and other factors that have potentially osteoblastogenic activity. In addition, our studies reveal that the active fraction that elicits these effects is in the <3 kD fraction in the *L. reuteri* secreted component.

## Discussion

In recent years growing evidence from murine models has highlighted the benefit of probiotic bacteria in treating adverse bone pathology associated with numerous conditions, including estrogen-deficiency and type 1 diabetes^11,13,16,17,34,49^. Furthermore, results from a recent randomized, double-blind placebo-controlled clinical trial have demonstrated that *L. reuteri* 6475 is able to reduce bone loss in older women^50^, further demonstrating its potential as a novel therapeutic. Although different probiotic bacteria have been shown to have beneficial effects on bone health, the complexity of host and bacterial mechanisms that mediate these effects are not well understood. Our lab has shown that treatment with the probiotic *L. reuteri* ATCC 6475 can enhance femoral trabecular bone volume fraction, bone mineral density and bone mineral content in intact healthy male mice^14^. Building upon these previous findings, in this study we have elucidated host as well as the bacterial mechanisms that enhance bone density in healthy male mice.

The intestine is residence to a significant population of immune cells that are in constant interaction with the intestinal microbiota. Immune cells present within the gut-associated lymphoid tissue (GALT), especially the mesenteric lymph nodes, play a critical role in inducing and maintaining tolerance to food proteins and commensal bacteria^51^; these immune cells can then re-enter the blood stream and circulate throughout the body^52^. Of the immune cells, T-lymphocytes are key players in maintaining the balance of bone remodeling and can exert an effect on both the bone-forming osteoblasts and the bone-resorbing osteoclasts. T cell-derived cytokines such as IL-10 and IL-17A have been demonstrated to inhibit/stimulate osteoclast differentiation as well as enhance mesenchymal stem cell proliferation and osteoblast differentiation^42–44,53^. These data suggest that intestinal probiotics could exert systemic bone effects through the modulation of T cell cytokine expression. In this context, we have identified that the presence of lymphocytes is crucial for the beneficial effect of *L. reuteri* on bone health. Consistent with our studies demonstrating that Rag KO mice do not respond to *L. reuteri* treatment, Dar *et al*. suggested that the effect of *L. acidophilus* in preventing OVX-induced bone loss is dependent on T-reg-Th17 balance^54^. Similarly, other probiotics such as LGG and the VSL#3 can decrease intestinal and bone marrow inflammation^49^ that could potentially benefit bone.

*L. reuteri* can influence the host through several mechanisms including modifying the gut microbiome composition and function as well as secreting factors that directly influence host cells. Even though our recent studies suggest that *L. reuteri* is able to alter the microbial communities in a model of ABX-induced dysbiosis^19^, taxonomy data suggest that under healthy conditions, *L. reuteri* does not modify broad, phylum level, bacterial community composition. This however, does not rule out changes in the proportion of specific bacterial species (especially low abundance species that are not sufficiently detected by 16 s sequencing) during/following *L. reuteri* treatment. Other studies have shown that healthy microbiota is resistant to significant changes in composition^55^ and that not all probiotics colonize the mouse and human gut^56,57^. In the current study, *L. reuteri* was administered orally at a dose of 3.3 × 10^8^ cfu/ml in the drinking water for 4-weeks. During transit, the bacteria feed on luminal nutrients and produce metabolites/factors which can cross the gut barrier. In fact, we have found significant differences in the serum profiles of mice treated with or without L. reuteri (unpublished data). Therefore, we examined direct effects of *L. reuteri* on the host (versus indirect effects via microbiota modifications). Consistent with previous findings^36^ we find that orally administered *L. reuteri* is able to translocate from the intestinal lumen to the mesenteric lymph nodes (not shown). Importantly, our findings also demonstrate that secreted products of *L. reuteri* can significantly affect MLN cytokine profile by increasing IL-10 expression and numbers of IL-10^+^ cells as well as other cytokines including TGFβ, IFNγ and IL-17A. Intriguingly we also find that both *L. reuteri* and its secreted components have immunomodulatory effects on CD4^+^ T cell cytokine profile and that these actions of *L. reuteri* on CD4^+^ T cells are negatively regulated by NOD signaling. In providing further evidence for a link between T-cell modulation of *L. reuteri* and effect on osteoblasts, we show that *L. reuteri* secreted products stimulate T-cells from MLNs to produce various factors (including cytokines) and these soluble factors from T-cells are able to modulate osteoblasts in culture. Although the precise identity of the factor(s) from MLN T-cells that stimulate(s) osteoblast gene expression is currently under investigation, taken together with the *in vivo* evidence from Rag knockout, our results support the possibility of *L. reuteri* secreted factors signaling MLN T-cells which subsequently contribute to the beneficial bone effects of *L. reuteri*. We recognize that our *in vitro* studies used factors produced by *L. reuteri* while grown in medium under cell culture conditions. These products may not necessarily be made by *L. reuteri* in the mouse intestine, which provides a different environment compared to a culture dish. Further studies are needed to identify the specific secreted factors and then test their ability to enhance bone health *in vivo*.

The most widely acknowledged paradigm of bacteria-immune cell interaction in the intestine involves the sampling of bacteria by dendritic cells and the presentation of these antigens to and subsequent programming of T cells^20^. Recent studies however, have suggested that bacteria can cross the intestinal barrier independent of APCs. In cirrhotic patients with ascites, increased bacterial DNA has been detected in the serum^58^ while in a murine model of social stress, DNA from commensal *Lactobacilli* have been detected in the spleen^59^. In addition to these disease models of bacterial translocation, a study by Schultz *et al*.^21^ demonstrated that a green fluorescent protein (GFP) probiotic E. coli strain Nissle 1917 was able to translocate from the intestinal lumen to the Peyer’s patches and MLNs in a time-dependent manner, with peak levels detected 6 hours post-gavage. Together, these data suggest that orally administered probiotics or their secreted products can potentially interact with cells in MLNs, and exert immunomodulatory effect via regulation of immune cells.

In this study, we examined the direct effect of *L. reuteri* on cells in the MLNs and find that several cytokines including IL-10 and IL-17 are significantly modulated by probiotic bacteria and its secreted products. This observed immunomodulatory effect of *L. reuteri* is comparable to that reported with other species of lactobacilli; *L. rhamnosus* Lcr35^60^, *L. casei*, *L. fermentum* Lb20, *L. plantarum* (Lb1 and 299 v), *L. johnsonii* La1^61^ and *L. gasseri* SBT2055^62^, have all been demonstrated to have concentration-dependent effects on dendritic cells. While the results from the whole MLN cultures suggested that the *L. reuteri* effects could be driven by APCs, the results from the CD3^+^ MLN cultures raised the possibility that these probiotic bacteria and secretory products could act directly on the T cells. This direct action on T cells was further confirmed in the CD4^+^ T cells from spleen where, as with the MLN cultures, *L. reuteri* had a concentration-dependent effect on cytokine expression. Perhaps the most interesting finding in this study however, is the discovery that soluble factors released by *L. reuteri* are able to modulate MLN and naïve CD4^+^ T cell cytokine profile at levels comparable to that observed in the whole live bacteria cultures. The identity of these molecules is currently under active investigation. This finding provides a potential mechanism by which oral administration of *L. reuteri* could have systemic effects and modulate T cells, which is critical for the beneficial bone effects as demonstrated in the Rag knockout mice.

Dendritic cells, as well as other immune cells, recognize components of bacteria known as pathogen-associated molecular pattern (PAMPs), using specific pattern-recognition receptors (PRRs) which are present on the host cell surface and in the cytosolic compartment^47,63^. Of the cell surface PRRs, the family of toll-like receptors (TLRs) have been demonstrated to respond to a large variety of PAMPs and activate the innate immune system. Of the cytosolic PRRs, the nucleotide binding and oligomerization domain (NOD)-like receptor family members NOD1 and NOD2 are the most well understood^64^. Studies have suggested that *Lactobacillus* species potentially interacts with the immune system and induces a response through TLR-2^45,62,65^, TLR-4 and DC-specific intracellular adhesion molecule 3-grabbing nonintegrin (DC-SIGN)^66^. Furthermore, studies utilizing NOD1 and NOD2 knockout mice have identified that these receptors play a key role in the regulation of bone mass by the intestinal microbiota^67^. Both TLR and NOD2 expression has been reported in T cells, suggesting that *L. reuteri* or its components could potentially act directly on the T cells, independent of APCs^68–70^. To understand the signaling mechanisms of how *L. reuteri* products activate T-cell cytokine expression, we therefore focused on TLR2 and NOD signaling pathways and our results are contrary to expectation in that the NOD signaling negatively affects *L. reuteri*-induced cytokine expression. While the physiological implication of this is unclear at this point, further extensive studies however are needed to delineate *L. reuteri*-stimulated signaling pathways in lymphocytes.

Probiotic bacteria, including lactobacilli, are known to secrete many factors that can potentially modulate the immune system including extracellular proteins^71^, short chain fatty acids (SCFA)^72^ and soluble peptides^73^. To try and identify the active molecules produced by *L. reuteri*, we fractionated the conditioned media. The active molecule was observed to be likely water soluble and within the <3kD fraction, suggesting either a non-protein molecule or a very small extracellular bacterial peptide. The characteristics of the *L. reuteri* active component in the present study had similar hallmarks to the active molecule(s) produced by the lactic acid bacteria *Bifidobacterium breve* and *Streptococcus thermophilus*^74^; a non-protein molecule <3kD in size. Interestingly, these authors suggest that the effects observed by *B. breve* and *S. thermophilus* may not be caused by the same active metabolite as some discordance was observed between experiments. Furthermore, the authors ruled out the SCFA butyrate and lactic acid as the metabolites responsible; as concentrations were too low and no inhibitory effect of LPS-induced TNFα secretion was observed at the levels present^74^. Butyrate is not a major component of lactobacilli fermentation; with different species and strains producing either very low levels or none at all^75–77^. In contrast, acetate is produced at much higher concentrations^76^. This could be of potential importance in suggesting a possible mechanism by which *L. reuteri* modulates T cell cytokine profile; treatment of CD4^+^ T cells upon initiation of differentiation with acetate has been demonstrated to promote IL-10 expression in all T cell polarized conditions (T helper (Th)17 and Th1) and non-polarized T cells, without affecting expression of Foxp3^78^.

In summary we have identified that the beneficial effects of the probiotic bacteria *L. reuteri* 6475 on bone are dependent on mature lymphocytes and that it is able to directly stimulate T-cells in the mesenteric lymph nodes and spleen. We also demonstrate that *L. reuteri* secretes active metabolites that are able to directly modulate CD4^+^ T cell cytokine profile and that this mechanism is negatively regulated by NOD signaling pathway. Furthermore, we have demonstrated that secreted factors from *L. reuteri* treated T cells can be beneficial to osteoblasts providing an important link between the effect of bacteria on lymphocytes and its beneficial bone effects. These findings highlight the potential mechanism by which *L. reuteri* is able to exert its beneficial systemic bone effect and highlight potential targets for future therapeutic research.

## Data Availability

The datasets generated during and/or analysed during the current study are available from the corresponding authors on reasonable request.
